# Insular threat associations within taxa worldwide

**DOI:** 10.1038/s41598-018-24733-0

**Published:** 2018-04-23

**Authors:** Camille Leclerc, Franck Courchamp, Céline Bellard

**Affiliations:** 1 Ecologie Systématique Evolution, Univ. Paris-Sud, CNRS, AgroParisTech, Université Paris-Saclay, 91400 Orsay, France; 2 Department of Genetics, Evolution and Environment, Centre for Biodiversity and Environment and Research, University College London, London, WC1E 6BT United Kingdom; 3 Unité Biologie des organismes et écosystèmes aquatiques (BOREA UMR 7208) Muséum national d’Histoire naturelle, Sorbonne Universités, Université Pierre et Marie Curie, Université de Caen Normandie, Université des Antilles, CNRS, IRD, 75005 Paris, France

## Abstract

The global loss of biodiversity can be attributed to numerous threats. While pioneer studies have investigated their relative importance, the majority of those studies are restricted to specific geographic regions and/or taxonomic groups and only consider a small subset of threats, generally in isolation despite their frequent interaction. Here, we investigated 11 major threats responsible for species decline on islands worldwide. We applied an innovative method of network analyses to disentangle the associations of multiple threats on vertebrates, invertebrates, and plants in 15 insular regions. Biological invasions, wildlife exploitation, and cultivation, either alone or in association, were found to be the three most important drivers of species extinction and decline on islands. Specifically, wildlife exploitation and cultivation are largely associated with the decline of threatened plants and terrestrial vertebrates, whereas biological invasions mostly threaten invertebrates and freshwater fish. Furthermore, biodiversity in the Indian Ocean and near the Asian coasts is mostly affected by wildlife exploitation and cultivation compared to biological invasions in the Pacific and Atlantic insular regions. We highlighted specific associations of threats at different scales, showing that the analysis of each threat in isolation might be inadequate for developing effective conservation policies and managements.

## Introduction

Global change has become a real concern over the last few decades, leading to a strong decrease in biodiversity and associated ecosystem services^1,2^. Among vertebrates, species populations have shown an average decline of 25% in abundance^3^. These trends are associated with exceptionally high rates of extinction, being around 1,000 times greater than historical rates^4,5^. In comparison to natural extinctions^6^, anthropogenic activities have played a significant role in recent extinctions on islands^7,8^. Although the specific mechanisms of extinctions linked to human disturbances are still debated, they involve several major threats such as habitat destruction, overexploitation, and introduction of alien species^9^. According to the International Union for the Conservation of Nature’s (IUCN) Red List of Threatened Species, over 80% of species are affected by more than one threat^10^. It is thus essential to consider all threats occurring at a global scale in order to gain a clearer picture of the causes of species decline worldwide and thus guide conservation actions, especially since they are likely to act in association^11^. However, investigating several threats covering both geographic and taxonomic scales from a worldwide perspective is a notoriously challenging task. Mostly due to data and computing limitations, studies exploring threats occurring at a global scale tend to investigate them individually^11,12^. In parallel, some studies have analyzed the effects of multiple threats, but they are restricted to specific geographic regions and delimited taxonomically, with a frequent bias for vertebrates^13–15^. Insular ecosystems, however, provide an excellent model system to tackle such an ambitious endeavor. First, islands are exceptionally rich reservoirs of biodiversity, as around one-quarter of all known extant vascular plant species are endemic to islands, for example, despite covering only 5% of the global land surface^16,17^. Second, such ecosystems due to their inherent characteristics (*e.g*., small population sizes, low habitat availability, low functional redundancy) are particularly vulnerable to the rapid anthropogenic changes of recent years, thus leading to an increased species extinction rate^18–20^.

Even with a good model system, the complex interplay between threats and species across space and time is challenging to investigate, because of limited techniques that simultaneously deal with three dimensions. Here, we tackle the complex species–threats–space system using network analyses^21,22^, considered to be a powerful approach for describing and exploring the complex interaction architecture between entities. This approach, recently applied to ecology, has generated numerous novel insights into the understanding of the structure, function, and dynamics of systems^21,22^.

Here, we applied network analyses to describe and analyze association patterns between biodiversity threats and extinct or threatened insular species. Species and threats are considered to be nodes, and connections between these two types of entities are established if a threat is responsible for the population decline of a given species. Using a comprehensive dataset of insular biodiversity and threats from the IUCN Red List^23^, we considered 11 categories of threat identified for more than 4,350 species of eight higher taxa distributed across 15 insular regions of the entire world. Identifying patterns of association between threats and species at macro-ecological scales provides unique insights into the layout of global threats across distinct taxa and specific regions, an important undertaking if we are to design appropriate actions to limit the current biodiversity crisis.

## Results

### Global scale

**Figure 1:**
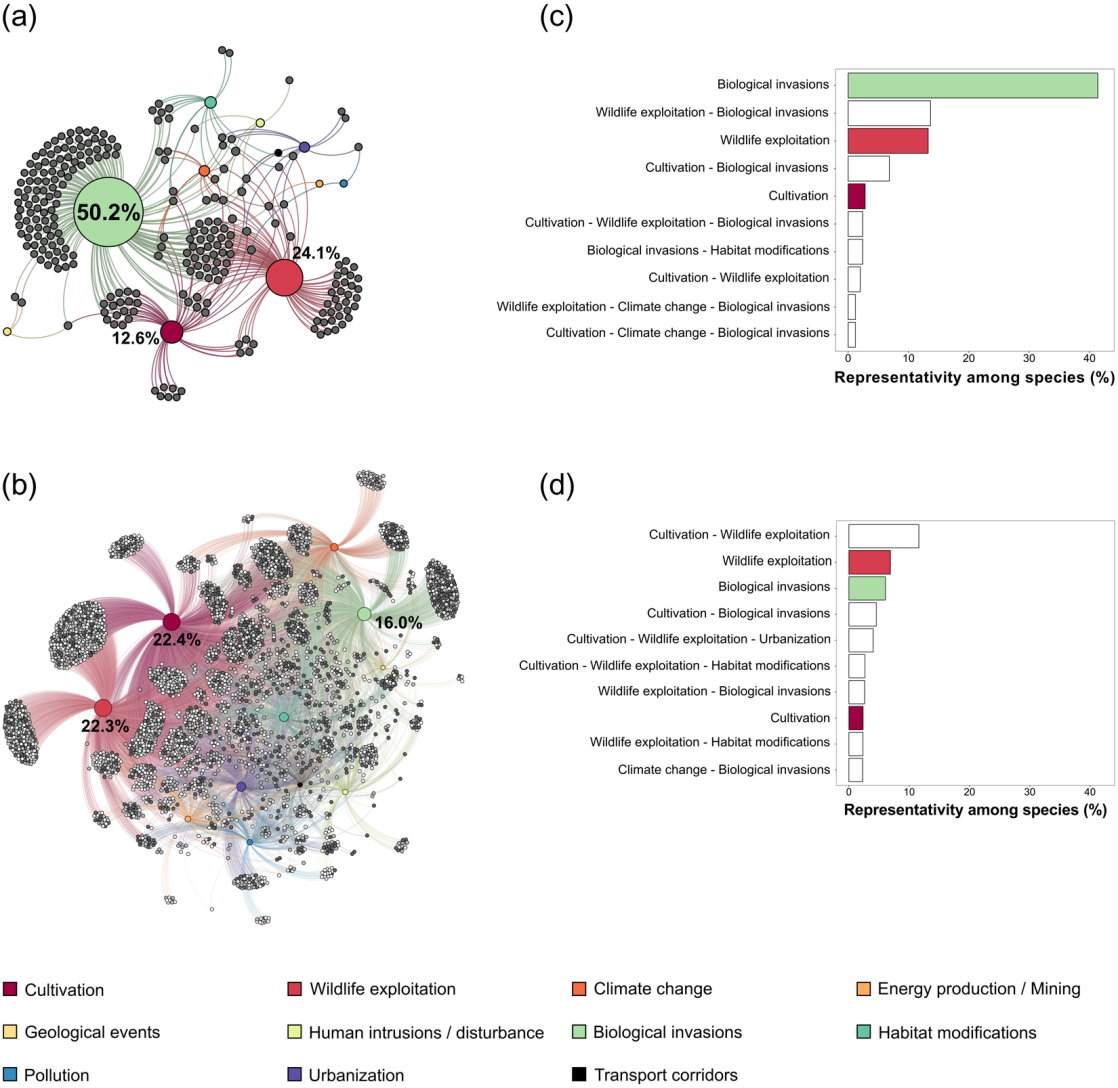
Graphical representation of species–threats interactions for (**a**) extinct species (n = 249) and (**b**) currently threatened species (n = 4,127) linked to the 11 threats. Colorful nodes reflect threats and gray nodes represent (**a**) extinct species or (**b**) species that are currently vulnerable (light gray), endangered (gray), and critically endangered (dark gray). Threat descriptions are given in Supplementary Table S4. The node size is proportional to their degree (*i.e*., number of interactions), and the percentage of the strongest interactions is indicated in the figure (see Supplementary Table S1 for further information). Top ten threats either acting alone or in association among (**c**) extinct (with 11 single threats and 23 threat associations) and (**d**) threatened species (with 11 single threats and 437 threat associations). Colorful bars reflect single threats, and white bars represent threat associations. Figures were created using Gephi 0.9.1 (https://gephi.org), R 3.3.1 (https://r-project.org), and Inkscape 0.91 (https://inkscape.org).

For extinct species, the species–threats network is formed by 260 nodes (*i.e*., 249 species and 11 threats) linked by 382 connections. Regarding the currently threatened species, the species–threats network links the 11 threats to 4,127 species through 10,530 connections (Fig. 1a,b). *Biological invasions* accounted for 50.2% of the total links in the extinct species network followed by *wildlife exploitation* (24.1%) and *cultivation* (12.6%) (Fig. 1a). These threats were also important for currently threatened species, but in a slightly different order: 22.4% for *wildlife exploitation*, 22.3% for *cultivation*, and 16.0% for *biological invasions* (Fig. 1b and Supplementary Table S1). Regarding the pattern of extinctions, these threats mostly acted alone (alone: 57.4% of total threats; in association: 40.1%), while they acted much more in association for threatened species (alone: 15.2%; in association: 75.4%) (Fig. 1c–d). Moreover, threats that are minor drivers of extinction and endangerment such as *habitat modifications*, *urbanization*, and *climate change* are often associated with one of the three major threats or with a set of these threats. For example, *urbanization* or *habitat modifications* associated with *cultivation* and *wildlife exploitation* accounted for 4.0% and 2.7% of the total current threats, respectively, while when acting alone, each accounted for around 1% (Fig. 1d). Compared to extinct species, threatened species faced more numerous threats, and the relative importance of these threats was more homogeneous. We observed a lower number of threats per extinct species (mean ± s.d.: 1.5 ± 0.6) than per threatened species (2.6 ± 1.0) (Supplementary Table S2). Also, threats such as *pollution* and *urbanization*, which are minor drivers of extinction, have gained in importance for threatened species. While *pollution* was a driver of extinction of two species (0.5% of total species), it was associated with 372 threatened species (3.5%) (Supplementary Table S1). Species at a higher risk of extinction (critically endangered > endangered > vulnerable) were not more associated with a given threat or with a higher number of threats (Fig. 1b and Supplementary Figure S1).

### Taxonomic scale

**Figure 2:**
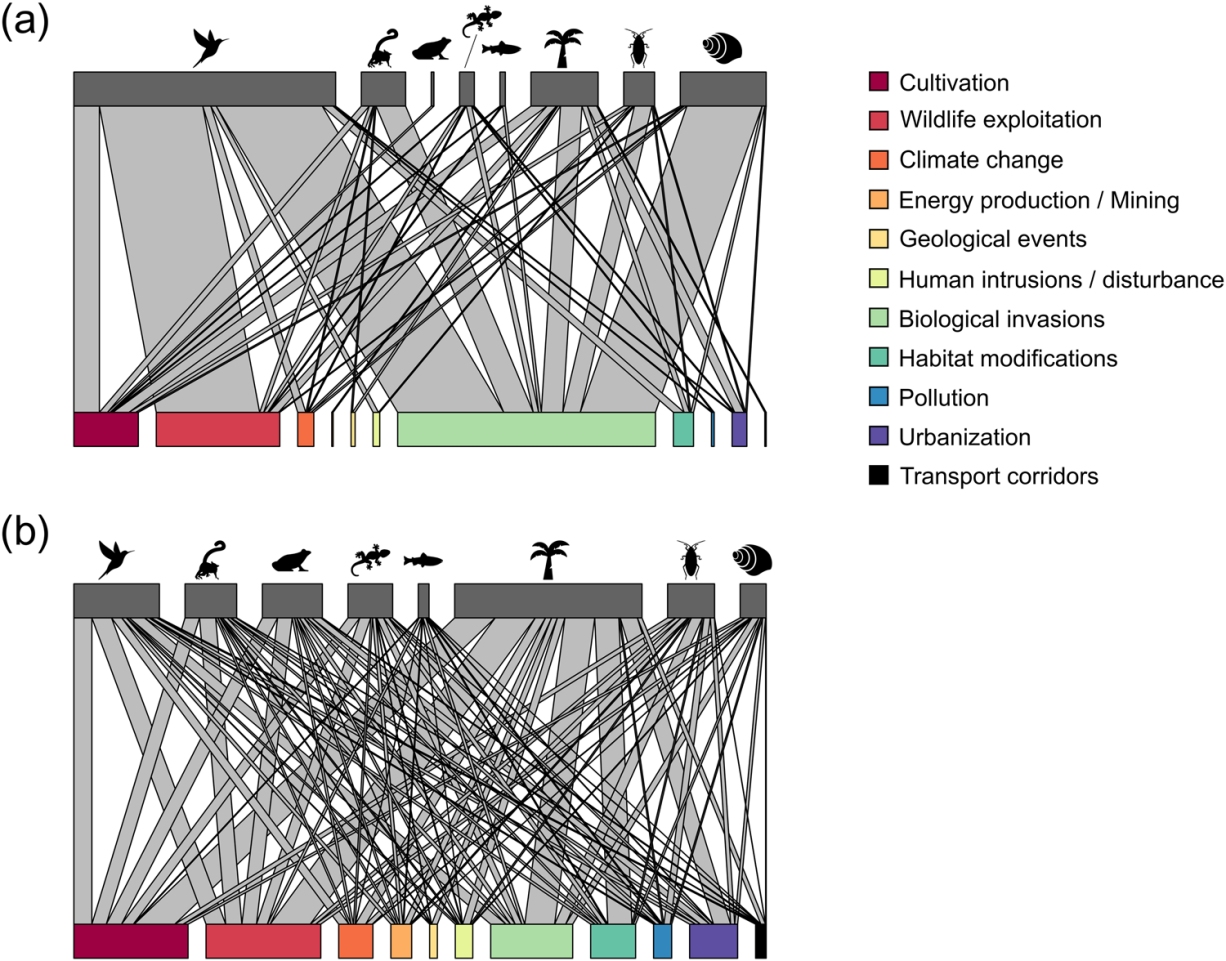
Graphical representation of networks connecting the proportion of threats (colored bars) to (**a**) extinct and (**b**) threatened species for eight taxa: birds, mammals, amphibians, reptiles, freshwater fish, plants, arthropods, and gastropods (top bars in gray). The width of links between taxa and threats is proportional to the sum of species–threat connections (see Supplementary Table S1 for further information). Figures were created using R 3.3.1 (https://r-project.org) and Inkscape 0.91 (https://inkscape.org). Icons made by Freepik from www.flaticon.com under a Flaticon Basic License.

Extinct species mostly included terrestrial vertebrates (58.6% including 46.6% birds, 9.2% mammals, 2.0% reptiles, and 0.8% amphibians) followed by invertebrates (29.7% including 23.7% gastropods and 6.0% arthropods), plants (10.5%), and freshwater fish (1.2%) (Supplementary Table S3). We found that *biological invasions* were the primary threat for extinct taxa, except for amphibians for which *cultivation* was the first and only threat (Fig. 2a, Supplementary Table S1). *Biological invasions* accounted for 79.3% of the total links in extinct invertebrates (comprising 15.0% arthropods and 64.3% gastropods), 50% for freshwater fish, 43.6% for terrestrial vertebrates (comprising 32.8% birds, 9.1% mammals, and 1.7% reptiles), and 32% for plants. *Wildlife exploitation* (terrestrial vertebrates: 34.5% comprising 31.9% birds, 1.3% mammals, 1.3% reptiles; plants: 14.0%) and *cultivation* (terrestrial vertebrates: 11.6% comprising 0.8% amphibians, 7.9% birds, 2.5% mammals, 0.4% reptiles; plants: 24.0%) were also highly connected to extinct terrestrial vertebrates and plants.

Threatened species mostly included terrestrial vertebrates (41.9% including 13.1% birds, 10.3% mammals, 9.7% amphibians, and 8.8% reptiles), followed by plants (39.7%), invertebrates (16.2% including 10.5% arthropods and 5.7% gastropods), and freshwater fish (2.2%). *Biological invasions* remained the main threat for invertebrates (24.9% of the total links, comprising 15.2% arthropods and 9.7% gastropods), and freshwater fish (27.1%), while *cultivation* and *wildlife exploitation* are mostly associated with plants and terrestrial vertebrates (Fig. 2b, Supplementary Table S1). Specifically, c*ultivation* accounted for 24.6% of the total links in terrestrial vertebrates (comprising 6.3% amphibians, 7.4% birds, 6.0% mammals, and 4.9% reptiles) and 21.6% for plants. Finally, *wildlife exploitation* accounted for 25.4% of the total links in terrestrial vertebrates (comprising 6.1% amphibians, 8.1% birds, 6.7% mammals, and 4.5% reptiles) and 21.4% for plants. The major threats are identical for threatened amphibians, birds, mammals, and reptiles, while differences appear regarding the minor threats (Supplementary Table S1). Overall, all threatened taxa faced more threats than extinct taxa. For example, five threats impacted extinct mammals, while all threats are associated with threatened mammals. In addition, extinct mammals were connected to fewer threats per species (mean ± s.d.: 1.4 ± 0.5) compared to threatened mammals (2.4 ± 1.1) (Supplementary Table S2).

### Spatial scale

**Figure 3:**
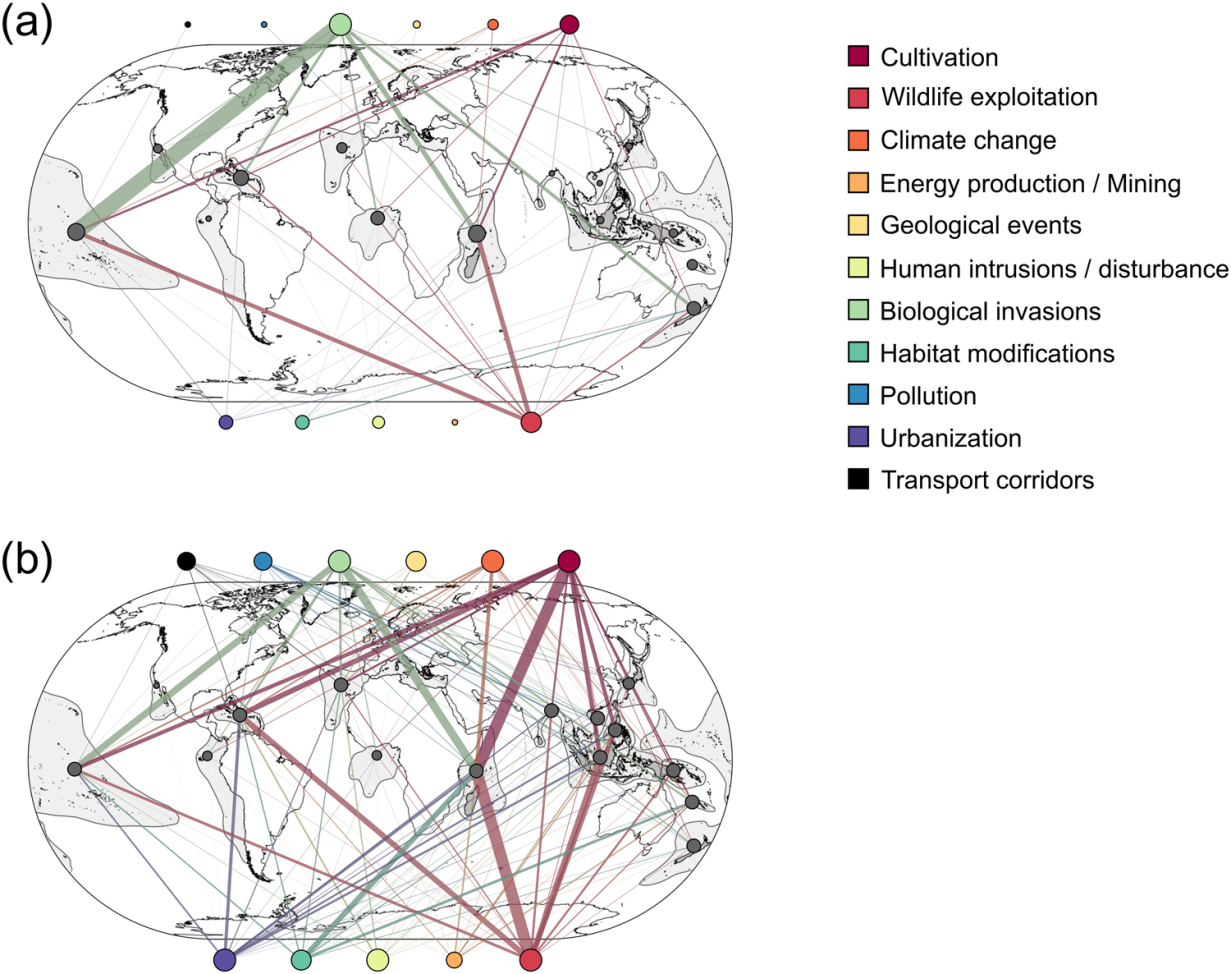
Graphical representation of networks illustrating species–threats interactions according to regions for (**a**) extinct and (**b**) threatened species. Colorful nodes reflect threats, and gray nodes represent insular regions. The node size is proportional to their degree (*i.e*., number of interactions), and the width of their respective links is proportional to the sum of species–threats connections. For the sake of clarity, threat nodes were placed outside the world map. Figures were created using Gephi 0.9.1 (https://gephi.org), QGIS 2.18.2 (https://qgis.org), and Inkscape 0.91 (https://inkscape.org).

Hotspots of extinction were located in *Polynesia and Micronesia* (43.4% of extinct species), *Madagascar* (22.1%), and *West Indies* (10.0%). The threat of *biological invasions* was a predominant driver of extinctions in *Polynesia and Micronesia*, *West Indies*, *New Zealand*, and *Madagascar* (Fig. 3a and Supplementary Figure S2). Also, *wildlife exploitation* was the second most important driver of extinctions across *Polynesia and Micronesia* and *Madagascar*.

Hotspots of current threats are also located in *Madagascar* (28.9% of threatened species), *West Indies* (13.3%), and *Polynesia and Micronesia* (11.2%) (Fig. 3b). The geographic distribution of threat importance highlighted a spatial pattern of threats. Indeed, *biological invasions* mainly threaten the Pacific and Atlantic insular regions (*i.e*., *Africa Atlantic*, *Mediterranean Basin*, *North America Pacific*, *Polynesia and Micronesia*, *South America Pacific*, except for the *West Indies*). The remaining insular regions, mostly located in the Indian Ocean and near the Asian coasts, are predominantly threatened by *wildlife exploitation* and *cultivation*. Threatened species within insular regions faced a higher number of threats, being on average 10.4 ± 0.88 compared to 4.0 ± 2.1 threats regarding the extinction pattern. For example, in *New Zealand*, six threats are associated with species extinction (threats per species: 2.0 ± 0.5), while all 11 threats currently impact threatened species (2.7 ± 1.0, Supplementary Table S2).

## Discussion

Our analysis is a first attempt to explore the relative importance of threats and their associations for species on islands. We showed that threatened species typically face numerous threats simultaneously (almost twice as many as extinct species). Clearly, *biological invasions*, *wildlife exploitation*, and *cultivation* have been responsible for the majority of insular extinctions (85%) and remain the main threats for insular biodiversity, impacting more than 60% of threatened species. Although the above three threats mostly acted alone regarding the pattern of extinctions, they tended more often to be combined, whether together or with other minor threats regarding the threatened species pattern. Such threat associations have already been observed as in the co-occurrence of invasions with harvesting or habitat loss (due to agriculture or aquaculture)^24^. Further, threats that were minor drivers of extinction risks such as *pollution* and *urbanization* have gained in importance by affecting a larger number of species, mainly in association with the three major threats. Thus, our results reaffirm the role of *biological invasions*, *wildlife exploitation*, and *cultivation* as major drivers of insular ecosystems losses^25,26^, analogous to those on the mainland^10,15^, but they also suggest the potential synergistic, antagonistic, or additive effects of threat interactions that may lead species to extinctions^27^.

Second, species at risk of extinction are mostly located in *Madagascar*, *West Indies*, and *Polynesia and Micronesia*, highlighting the impact even in less populated and accessible areas. Nevertheless, this result is dependent on sampling. Indeed, a region with a high sampling effort is more likely to present a greater number of identified species and so be a hotspot of endangered species. All insular regions were shown to suffer from a high number of threats, but the relative importance of threats across regions shows a strong East-West pattern. Indeed, threatened species on the Pacific and Atlantic islands suffer primarily from *biological invasions*, while species in the Indian Ocean and near the Asian coasts are predominantly threatened by *wildlife exploitation* and *cultivation*. The Pacific and Atlantic regions have suffered from different waves of colonization (*i.e*., Polynesian discovery followed by European colonization), which have strongly contributed to the introduction of invasive species^20,28^ and are likely to explain the predominance of the *biological invasions* threat for extinct and threatened species throughout these regions. Nevertheless, most studies on invasive species impacts have focused on the Pacific region^29^, while invasions in Asia (mainland and insular) are understudied^30^. At the same time, a large proportion of tropical forests are located in Southeast Asia^31,32^. These forests are highly threatened by rapid deforestation due to, for example, agricultural expansion or commercial logging caused by the exploding human demography^33,34^. It has also been shown that overexploitation is the most prominent threat within Asia, where harvesting for food and use in traditional Chinese medicine are the two main forms of overexploitation^15^. The relative importance of threats among and between insular regions could also be partly explained by island biogeography. Indeed, some insular parameters (*e.g*., area, isolation) have been linked to species vulnerability to threats^19^.

Third, all taxa suffer from several simultaneous threats, which may vary in importance within taxa. For example, *wildlife exploitation* and *cultivation* were mostly associated with threatened plants and terrestrial vertebrates, while *biological invasions* were mainly responsible for threatened invertebrates and freshwater fish. Several studies have already documented the impact of overexploitation, agriculture/aquaculture, and invasive species on terrestrial vertebrates and plants^35–37^, and our results confirm their major role as a driver of species extinction and decline. The role of these threats as drivers of invertebrate and freshwater fish losses is less documented^38^. Our results provide a first global insight into these taxonomic groups and their associated threats at different macro-ecological scales, but further research is clearly necessary. The relative importance of threats among and between taxa could be due not only to differences in species’ exposure to threats but also to species’ sensitivity. Some species can be more vulnerable to a specific threat than others due to biotic (*e.g*., species’ traits) and abiotic factors (*e.g*., species’ environment)^39,40^. For example, mammal families composed of small-size habitat specialists are more likely to be threatened by habitat-modifying processes^39^. The interaction between species’ traits and threat vulnerability can be synergistic. For example, such an interaction was observed between body mass and hunting vulnerability in Chinese birds^41^. Also, to explain the differences in threats to extinct versus threatened species, two explanations can be proposed. First, it could be due to differences in information, with historical knowledge lacking for some extinct species (see below). Second, it could also be associated with the vulnerability of species, whereby the species most vulnerable to certain threats would be first to become extinct, thus leaving a greater number of less vulnerable species, as has been shown for birds and biological invasions^19,20^.

Although the IUCN Red List is the most comprehensive assessment of species that are at risk of extinction worldwide^42,43^, this dataset has several shortcomings. Firstly, small species with narrow distribution ranges and low dispersal abilities, which are mostly local endemics, are underassessed^44^. IUCN has successfully completed an assessment of the threat status of birds, mammals, and the vast majority of described amphibians, while global assessments for other taxa are yet to be properly documented^44,45^. Taxonomic gaps imply that our results relating to plants, invertebrates, and freshwater fish should be taken with more caution than those of terrestrial vertebrates. Second, recent studies have pointed out that the application of Red List criteria generally tends to overestimate extinction risks for most island endemics, which naturally have very small areas of occupancy and extents of occurrence, even if they are common within their range^46,47^. However, when we restricted our analyses to the 777 threatened species significantly and harshly impacted by threats, a similar pattern was observed, and importantly, it did not alter our conclusions (Supplementary Figure S3). Third, despite showing an apparent difference in threats between extinct and threatened species, this result could be due to the fact that attributing a cause to species extinction and endangerment is difficult. Indeed, the attribution of extinction causes is based on historical knowledge, for which little written information is available, especially for species that disappeared more than a century ago^7^. Further, only the most likely causes for extinction are sometimes assigned. Last we only assessed species richness on species–threats associations, and despite being the most commonly used measure of diversity in macro-ecology, this metric does not consider the evolutionary and functional differences among species^48^.

In spite of these limitations, our study is a first step to investigate mechanisms, processes, and drivers affecting species–threats associations or threat interactions in the future. Our findings reveal that investigating threats in isolation is insufficient when exploring patterns and so defining effective conservation policies and managements, as specific associations of threats affect the different levels of species, taxa, and region. Today, large-scale conservation planning such as the protection of biodiversity hotspots^49^ has been widely recognized as crucial for guiding global conservation investments, but this conservation strategy only relies on high rate of endemism and high rates of habitat loss. Our study considers 11 different threats to identify where insular biodiversity is the most endangered worldwide and which specific threats are responsible for such a pattern. Specifically, we argue that *Madagascar*, *West Indies*, and *Polynesia and Micronesia* should be considered to be the top priorities to preserve insular taxonomic diversity worldwide by undertaking specific actions associated with *biological invasions*, *wildlife exploitation*, and *cultivation*. In fact, our approach allows us to identify broad guidelines such as extending the protected areas already set up in the *West Indies*, which are mostly impacted by habitat loss and degradation. However, more case-oriented studies at local scales of species–threats associations should be undertaken in order to set effective conservation guidelines.

## Materials and Methods

### IUCN Red List Database

The IUCN Red List of Threatened Species is widely regarded as the world’s most comprehensive information source on the global extinction risk of species^42,43^. It is used in a varied way: setting priorities in the compilation of species action plans, reserve selection, and management, or defining indicators for the state of the environment^50–52^. The IUCN Red List^23^ and BirdLife International^53^ have assessed the conservation status of more than 12,680 insular terrestrial and freshwater species. In this study, we focused our analyses on the 15 most documented insular regions worldwide (*i.e*., containing >50 assessed species) (Supplementary Figure S2; *Africa Atlantic*, *Asian Coast*, *East Indies*, *Indo-Burma*, *Japan*, *Madagascar*, *Mediterranean Basin*, *New Caledonia*, *New Zealand*, *North America Pacific*, *Papua New Guinea*, *Philippines*, *Polynesia and Micronesia*, *South America Pacific*, and *West Indies*), which together encompass 12,533 insular species. We restricted our analyses to species that have been classified as extinct (*i.e*., extinct and extinct in the wild; n = 450) and at risk of extinction (*i.e*., vulnerable, endangered, and critically endangered; n = 6,026). In addition, the IUCN Red List has assessed and classified threats impacting these species through the IUCN threat classification scheme (version 3.2)^54^. In our study, we considered 11 classes of major threats (Supplementary Table S4). Based on the threat categories, the causes of species decline were identified for 249 extinct and 4,127 threatened species. These insular species belong to eight higher taxa (hereafter, taxa): 402 amphibians, 451 arthropods, 655 birds (resident breeding), 93 freshwater fish, 293 gastropods, 449 mammals, 1,665 plants, and 368 reptiles (Supplementary Table S3). Furthermore, additional threat information was recorded such as the timing of the threat (past, ongoing, future, or unknown), its scope (*i.e*., proportion of the total population affected), and its severity (*i.e*., overall declines caused by the threat). Using this supplementary information, we identified 777 threatened insular species as being significantly and harshly impacted by threats (*i.e*., scope: whole (>90%) and majority (50–90%) of the population impacted; severity: very rapid, rapid, and slow significant population declines).

### Bipartite networks

At the most basic level, a network is a set of entities called nodes connected by edges. Specifically, bipartite networks are a type of network where nodes are partitioned into two distinct types (here, insular species and threats) and interactions occur exclusively between types. Because the relationships between insular species and threats are complex (*i.e*., species are associated with multiple threats, while each of the 11 threats impacts several species), we used three separate networks to analyze species–threats associations at the species, taxa, and then geographic levels. We also investigated each of the three network scales separately for extinct and threatened species.

#### Networks between insular species and threats

First, we built unweighted bipartite networks to analyze the threats associated with species’ extinctions and risks of extinction. Networks were created with R software (version 3.3.1)^55^ using *igraph* and *rgexf* packages^56,57^. Then, network structures were explored and visualized with the interactive platform Gephi^58^ using a *ForceAtlas 2* layout. A layout is an algorithm that positions nodes in a 2-D or 3-D space. *ForceAtlas 2* layout^59^ allows the network to be visualized based on the forces of attraction and repulsion, where the nodes repulse each other based on their degree (*i.e*., the number of incoming and outgoing edges), while the edges attract the connected nodes. As a result, high-degree nodes tend to separate from other high-degree nodes and attract low-degree nodes to which they are connected. In our case, species that tend to be associated with the same threat or group of threats will be clustered and repulsed from other threats and/or species that are associated with different threats.

#### Networks between taxa and threats

Second, we built weighted bipartite networks to analyze the associations to threats at the taxa level. Each taxa was connected to threats with an associated weight depending on the number of their species threatened by each threat. Networks were created and visualized using the *plotweb* function implemented in the R package *bipartite*^60^.

#### Networks between insular regions and threats

Third, we built weighted bipartite networks to analyze insular regions–threats associations. Each of the 15 insular regions was connected to threats with an associated weight depending on the number of insular species threatened by each threat in that region. As above, networks were created using the R packages *igraph* and *rgexf*, and network structures were explored and visualized with Gephi software using a *Geo Layout*, which uses latitude and longitude coordinates to set the node position in the graphic space.

For each of the three network types, we estimated the number of species associated with each threat as well as the number of threats per species. Analyses were performed using Gephi software as well as with some packages of R software (version 3.3.1): *bipartite*, *igraph*, *rgexf*, *ggplot2*^61^, *dplyr*^62^ and *plyr*^63^.

## Electronic supplementary material

Supplementary Information
