# Supplementary material for: Insular threat associations within taxa worldwide
Source: Sci Rep. 2018 Apr 23;8:6393. doi: 10.1038/s41598-018-24733-0 (PMC5913315; doi:10.1038/s41598-018-24733-0)
Supplement: Supplementary file 1 — Supplementary Information [file 41598_2018_24733_MOESM1_ESM.pdf]

Insular threat associations within taxa worldwide

C. Leclerc, F. Courchamp & C. Bellard

Supplementary Information contains:

- Four Supplementary Tables:

Supplementary Table S1. Number (and percentage) of species associated to each threat according to extinct and threatened species networks

Supplementary Table S2. Mean and standard deviation of the number of species associated to each threat and of the number of threat per species

Supplementary Table S3. Number of extinct and threatened species recorded by IUCN Red List having information about threats

Supplementary Table S4. The 11 classes of major threats from the IUCN Red List, with associated definition

- Three Supplementary Figures:

Supplementary Figure S1. Violin plot of threats' number according vulnerable, endangered and critically endangered species

Supplementary Figure S2. Map of the 15 large insular regions used in this study

Supplementary Figure S3. Graphical representation of threatened species–threats interactions

Supplementary Table S1 │ Number (and percentage in brackets) of species associated to each threat according to extinct and threatened species networks (see Figure 1 and Figure 2)**.**

	Extinct species–threats network									Threatened species–threats network								
	**Global**	**Birds**	**Mammals**	**Amphibians**	**Reptiles**	**Freshwater fishes**	**Plants**	**Arthropods**	**Gastropods**	**Global**	**Birds**	**Mammals**	**Amphibians**	**Reptiles**	**Freshwater fishes**	**Plants**	**Arthropods**	**Gastropods**
Cultivation	48 (12.6)	19 (9.7)	6 (18.2)	2 (100)	1 (9.1)	1 (25.0)	12 (24.0)	5 (21.8)	2 (3.1)	2,345 (22.3)	369 (21.0)	297 (28.0)	313 (25.4)	243 (26.5)	18 (8.1)	832 (21.6)	172 (17.8)	101 (18.9)
Wildlife exploitation	92 (24.1)	77 (39.5)	3 (9.1)	--	3 (27.3)	1 (25.0)	7 (14.0)	--	1 (1.6)	2,356 (22.4)	404 (23.0)	332 (31.3)	300 (24.4)	224 (24.4)	57 (25.8)	822 (21.4)	110 (11.4)	107 (20.1)
Climate change	12 (3.1)	6 (3.1)	1 (3.0)	--	1 (9.1)	--	--	2 (8.7)	2 (3.1)	702 (6. 7)	210 (12.0)	44 (4.1)	48 (3.9)	37 (4.0)	3 (1.4)	210 (5.5)	137 (14.2)	13 (2.4)
Energy production & Mining	1 (0.3)	--	--	--	--	--	1 (2.0)	--	--	428 (4.0)	93 (5.3)	46 (4.3)	43 (3.5)	66 (7.2)	15 (6.8)	127 (3.3)	35 (3.6)	3 (0.6)
Geological events	3 (0.8)	--	1 (3.0)	--	--	--	2 (4)	--	--	158 (1.5)	14 (0.8)	14 (1.3)	5 (0.4)	8 (0.9)	--	117 (3.0)	--	--
Human intrusions / disturbance	5 (1.3)	4 (2.1)	--	--	--	--	--	1 (4.3)	--	359 (3.4)	48 (2.7)	34 (3.2)	52 (4.2)	15 (1.6)	2 (0.9)	155 (4.0)	35 (3.6)	18 (3.4)
Biological invasions	192 (50.2)	79 (40.5)	22 (66.7)	--	4 (36.3)	2 (50.0)	16 (32.0)	13 (56.6)	56 (87.5)	1,684 (16.0)	313 (17.8)	95 (9.0)	95 (7.7)	136 (14.8)	60 (27.1)	613 (15.9)	227 (23.5)	145 (27.2)
Habitat modifications	15 (3.9)	8 (4.1)	--	--	--	--	4 (8.0)	1 (4.3)	2 (3.1)	923 (8.8)	93 (5.3)	69 (6.5)	62 (5.0)	104 (11.3)	24 (10.9)	495 (12.9)	30 (3.1)	45 (8.6)
Pollution	2 (0.5)	1 (0.5)	--	--	1 (9.1)	--	--	--	--	372 (3.5)	37 (2.1)	16 (1.5)	93 (7.5)	9 (1.0)	29 (13.1)	28 (0.7)	131 (13.6)	29 (5.4)
Urbanization	11 (2.9)	1 (0.5)	--	--	1 (9.1)	--	7 (14.0)	1 (4.3)	1 (1.6)	986 (9.4)	123 (7.0)	98 (9.2)	192 (15.6)	65 (7.1)	12 (5.4)	352 (9.2)	84 (8.7)	60 (11.3)
Transport corridors	1 (0.3)	--	--	--	--	--	1 (2.0)	--	--	217 (2.0)	52 (3.0)	16 (1.5)	29 (2.4)	10 (1.1)	1 (0.4)	95 (2.5)	3 (0.3)	11 (2.1)

Supplementary Table S2│Mean and standard deviation of the number of species associated to each threat and of the number of threat per species according three scales: Global (Figure 1), Taxonomic (Figure 2) and Geographic (Figure 3).

	**Extinct**		**Threatened**	
	Number of species associated to each threat (mean ± sd)	*Number of threats per species* (mean ± sd)	*Number of species associated to each threat* (mean ± sd)	*Number of threats per species* (mean ± sd)
Global	34.7 ± 41.4	1.5 ± 0.6	957.3 ± 644.0	2.6 ± 1.0
Invertebrates	12.4 ± 16.2	1.2 ± 0.3	149.7 ± 84.7	2.2 ± 0.8
Arthropods	3.8 ± 4.7	1.5 ± 0.7	96.4 ± 71.9	2.2 ± 1.0
Gastropods	10.6 ± 22.2	1.0 ± 0.3	53.3 ± 48.8	2.2 ± 0.9
Freshwater fishes	1.3 ± 0.4	1.3 ± 0.4	22.1 ± 16.3	2.5 ± 1.1
Plants	6.3 ± 4.3	1.9 ± 0. 6	349.6 ± 248.3	2.3 ± 1.0
Terrestrial Vertebrates	26.8 ± 30.1	1.7 ± 0.6	451.5 ± 326.0	2.9 ± 1.0
Birds	24.3 ± 33.5	1.6 ± 0.7	159.6 ± 141.4	3.2 ± 1.3
Mammals	6.6 ± 8.8	1.4 ± 0.5	96.4 ± 111.9	2.4 ± 1.1
Amphibians	2 ± 0.0	1 ± 0.0	112.0 ± 107.7	3.0 ± 1.2
Reptiles	1.8 ± 1.3	2.2 ± 0.8	83.3 ± 85.1	2.5 ± 1.1
Africa Atlantic	4.3 ± 3.1	1.7 ± 0.6	20.9 ± 13.0	2.4 ± 1.1
Asian Coast	1.0 ± 0.0	1.0 ± 0.0	18.0 ± 15.6	2.2 ± 0.9
East-Indies	1.0 ± 0.0	2.0 ± 0.0	100.2 ± 82.6	2.6 ± 1.0
Indo-Burma	2.0 ± 0.0	1.0 ± 0.0	48.9 ± 42.1	3.0 ± 1.1
Japan (and the East Sea Islands)	3.5 ± 0.5	1.8 ± 0.4	22.7 ± 10.7	3.0 ± 1.2
Madagascar (and the Indian Ocean Islands)	10.5 ± 10.1	1.5 ± 0.7	252.7 ± 225.3	2.3 ± 0.9
Mediterranean Basin	1.8 ± 0.8	2.3 ± 0.9	69.8 ± 25.7	2.9 ± 1.1
New Caledonia	1.3 ± 0.4	2.5 ± 0.5	57.1 ± 44.3	3.3 ± 0.8
New Zealand	7.7 ± 4.9	2.0 ± 0.5	25.1 ± 14.6	2.7 ± 1.0
North America Pacific	2.6 ± 1.6	1.3 ± 0.4	6.6 ± 5.8	1.5 ± 0.7
Papua New Guinea	1.3 ± 0.4	2.0 ± 1.0	30.0 ± 26.7	2.2 ± 0.9
Philippines	--	--	77.9 ± 63.3	2.8 ± 0.9
Polynesia and Micronesia	18.8 ± 21.9	1.4 ± 0.5	108 ± 88.4	2.5 ± 1.0
South America Pacific	2.0 ± 0.0	1.0 ± 0.0	17.1 ± 14.8	2.3 ± 0.7
West Indies	5.4 ± 4.5	1.5 ± 0.5	127.9 ± 86.1	2.5 ± 1.0

Supplementary Table S3 | Number of extinct and threatened species recorded by IUCN Red List having information about threats.

	Extinct species	Threatened species
Terrestrial Vertebrates	146	1,728
Amphibians	2	400
Birds	116	539
Mammals	23	426
Reptiles	5	363
Plants	26	1,639
Invertebrates	74	670
Arthropods	15	436
Gastropods	59	234
Freshwater fishes	3	90
**Total**	**249**	**4,127**

Supplementary Table S4 │The 11 classes of major threats from the IUCN Red List, with associated definition. In brackets, the original threat names from Salafsky *et al.* (2008).

**IUCN - CMP Unified Classification of Direct Threats**	
Cultivation *(agriculture & aquaculture)*	Threats from farming and ranching as a result of agricultural expansion and intensification, including silviculture, mariculture and aquaculture (includes the impacts of any fencing around farmed areas).
Wildlife exploitation (*biological resource use*)	Threats from consumptive use of "wild" biological resources including both deliberate and unintentional harvesting effects; also persecution or control of specific species.
Climate change *(climate change & severe weather*)	Threats from long-term climatic changes, which may be linked to global warming and other severe climatic/weather events that are outside of the natural range of variation, or potentially can wipe out a vulnerable species or habitat.
Energy production & Mining	Threats from production of non-biological resources.
Geological events	Threats from catastrophic geological events.
Human intrusions & disturbance	Threats from human activities that alter, destroy and disturb habitats and species associated with non-consumptive uses of biological resources.
Biological Invasions (*invasive & other problematic species, genes & diseases*)	Threats from non-native and native plants, animals, pathogens/microbes, or genetic materials that have or are predicted to have harmful effects on biodiversity following their introduction, spread and/or increase in abundance.
Habitat modifications *(natural system modifications)*	Threats from actions that convert or degrade habitat in service of "managing" natural or semi-natural systems, often to improve human welfare.
Pollution	Threats from introduction of exotic and/or excess materials or energy from point and nonpoint sources.
Urbanization (*residential & commercial development*)	Threats from human settlements or other non-agricultural land uses with a substantial footprint.
Transport corridors *(transportation & service corridors)*	Threats from long narrow transport corridors and the vehicles that use them including associated wildlife mortality.

Salafsky, N. *et al.* A standard lexicon for biodiversity conservation: Unified classifications of threats and actions. *Conserv. Biol.* **22,** 897–911 (2008).

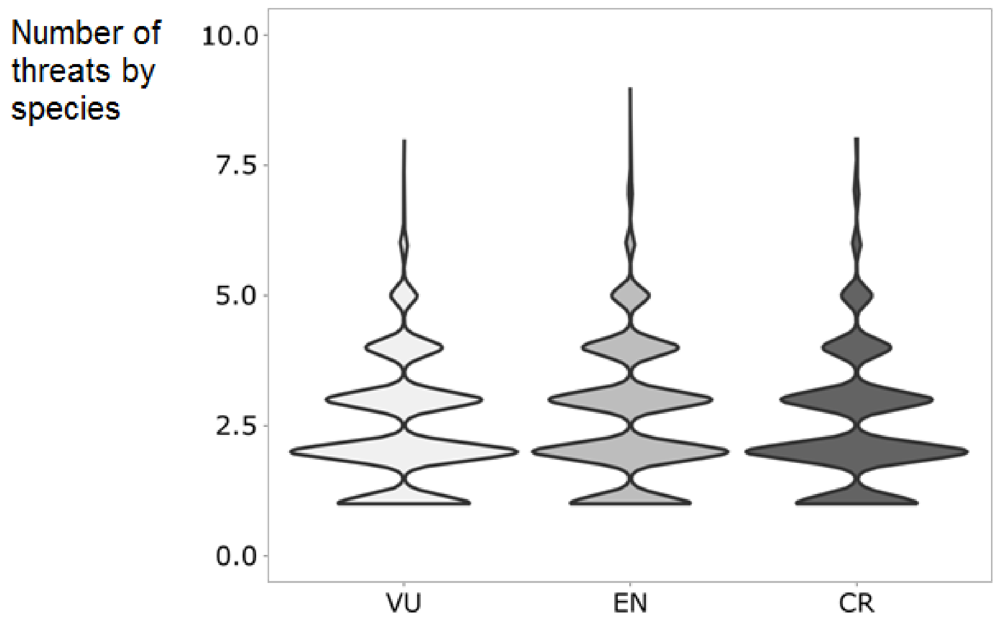

Supplementary Figure 1│Violin plot (showing the probability density of the data at different values) of threats' number according vulnerable (VU), endangered (EN) and critically endangered (CR) species. Figure was created using R 3.3.1 (https://r-project.org).

We perform a Kruskal-Wallis test, because our data do not follow a normal distribution, for testing whether samples originate from the same distribution. We used the kruskal.test() function implemented in the R package *stats*.

Test result:

Kruskal-Wallis chi-squared = 4.973, df = 2, p-value = 0.083

The conclusion is therefore that the means of the three endangerment categories (VU, EN, CR) are statistically equal.

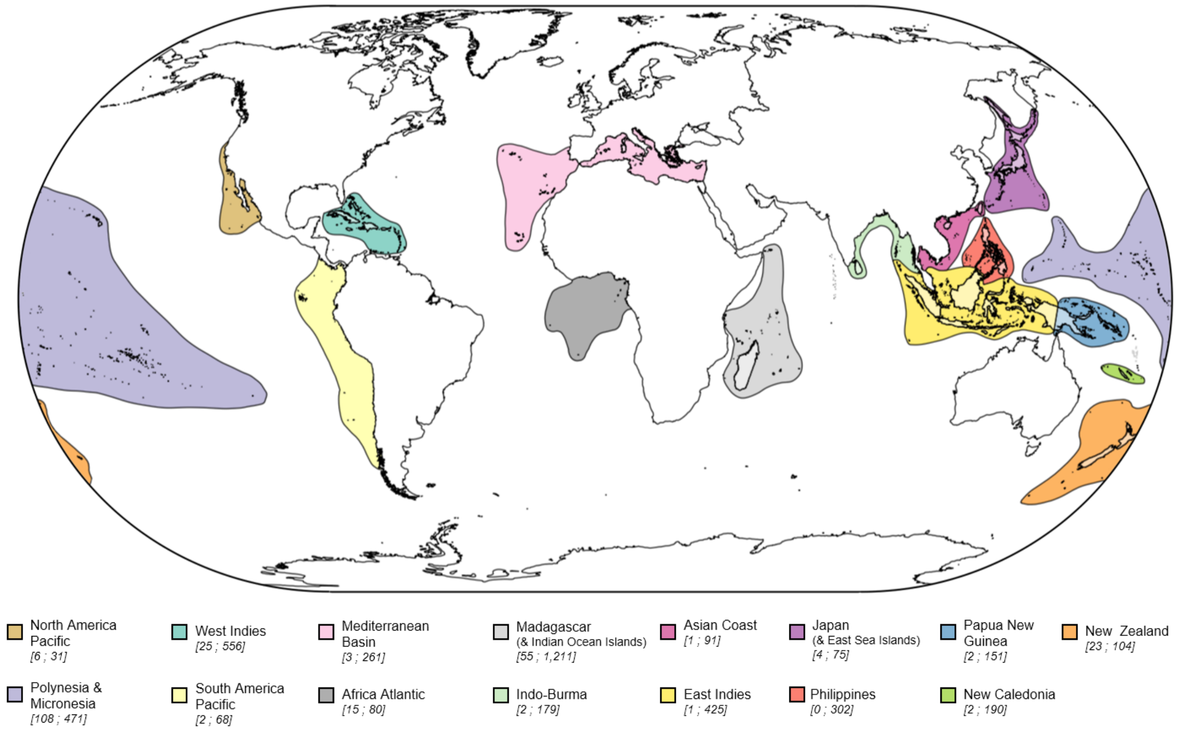

Supplementary Figure S2│Map of the 15 large insular regions used in this study. Numbers in brackets refer respectively to extinct and threatened species in each insular region. Figure was created using QGIS 2.18.2 (https://qgis.org) and Inkscape 0.91 (https://inkscape.org).

	Degree metric
Cultivation	284 (21.1%)
Wildlife exploitation	338 (25.1%)
Climate change	108 (8.0%)
Energy production & Mining	50 (3.7%)
Geological events	12 (0.9%)
Human intrusions / disturbance	21 (1.6%)
Biological invasions	308 (22.9%)
Habitat modifications	105 (7.8%)
Pollution	41 (3.1%)
Urbanization	56 (4.2%)
Transport corridors	21 (1.6%)

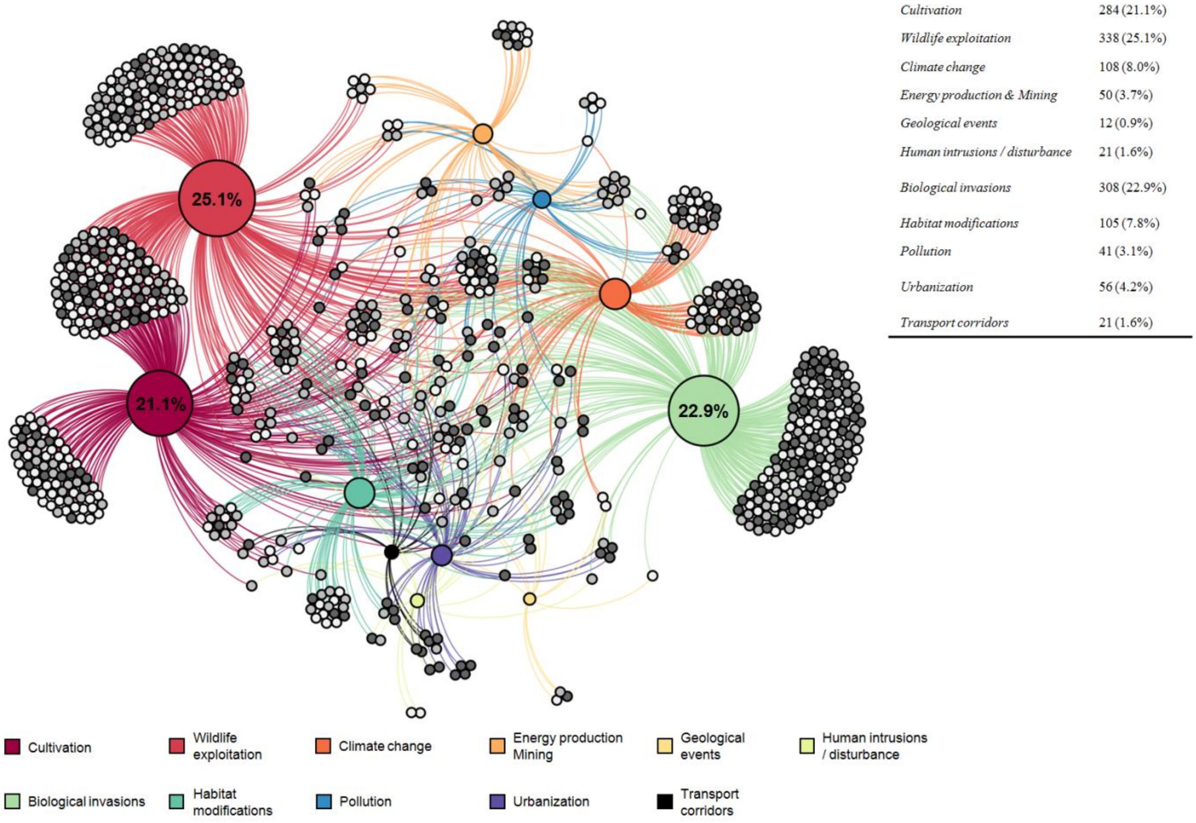

Supplementary Figure S3 | Graphical representation of threatened species (n=777) – threats (n=11) interactions. Only threatened species impacted by threats (*i.e.*, whole >90% and majority 50–90% population impacted; and very rapid, rapid and slow significant declines of population) were taken into account in the network, representing 18.8% of all threatened species (VU, EN, CR). Colorful nodes reflect threats and grey nodes represent species that are currently vulnerable (light grey), endangered (grey) and critically endangered (dark grey). Description of threats are given in Supplementary Table 1. The nodes size is proportional to their degree (*i.e.*, number of interactions) and the percentage of the strongest interactions is indicated on the figure. Figure was created using Gephi 0.9.1 (https://gephi.org), R 3.3.1 (https://r-project.org), and Inkscape 0.91 (https://inkscape.org).
